# Rapid draft sequencing and real-time nanopore sequencing in a hospital outbreak of *Salmonella*

**DOI:** 10.1186/s13059-015-0677-2

**Published:** 2015-05-30

**Authors:** Joshua Quick, Philip Ashton, Szymon Calus, Carole Chatt, Savita Gossain, Jeremy Hawker, Satheesh Nair, Keith Neal, Kathy Nye, Tansy Peters, Elizabeth De Pinna, Esther Robinson, Keith Struthers, Mark Webber, Andrew Catto, Timothy J. Dallman, Peter Hawkey, Nicholas J. Loman

**Affiliations:** Institute of Microbiology and Infection, University of Birmingham, Birmingham, B15 2TT UK; NIHR Surgical Reconstruction and Microbiology Research Centre, University of Birmingham, Birmingham, B15 2TT UK; Public Health England, Colindale, London, UK; Public Health England, Field Epidemiology Service (Birmingham Office), Birmingham, UK; Public Health England Birmingham Public Health Laboratory, Heart of England NHS Trust, Birmingham, UK; Department of Microbiology, University of Warwick, Warwick, UK; Medical Directorate, Heart of England NHS Trust, Birmingham, UK

## Abstract

**Background:**

Foodborne outbreaks of *Salmonella* remain a pressing public health concern. We recently detected a large outbreak of *Salmonella enterica* serovar Enteritidis phage type 14b affecting more than 30 patients in our hospital. This outbreak was linked to community, national and European-wide cases. Hospital patients with *Salmonella* are at high risk, and require a rapid response. We initially investigated this outbreak by whole-genome sequencing using a novel rapid protocol on the Illumina MiSeq; we then integrated these data with whole-genome data from surveillance sequencing, thereby placing the outbreak in a national context. Additionally, we investigated the potential of a newly released sequencing technology, the MinION from Oxford Nanopore Technologies, in the management of a hospital outbreak of *Salmonella*.

**Results:**

We demonstrate that rapid MiSeq sequencing can reduce the time to answer compared to the standard sequencing protocol with no impact on the results. We show, for the first time, that the MinION can acquire clinically relevant information in real time and within minutes of a DNA library being loaded. MinION sequencing permits confident assignment to species level within 20 min. Using a novel streaming phylogenetic placement method samples can be assigned to a serotype in 40 min and determined to be part of the outbreak in less than 2 h.

**Conclusions:**

Both approaches yielded reliable and actionable clinical information on the *Salmonella* outbreak in less than half a day. The rapid availability of such information may facilitate more informed epidemiological investigations and influence infection control practices.

**Electronic supplementary material:**

The online version of this article (doi:10.1186/s13059-015-0677-2) contains supplementary material, which is available to authorized users.

## Background

Outbreaks of *Salmonella* from contaminated food are frequently reported in the community, with 1.2 million cases estimated to occur in the US each year [1]. In a population-based study in the UK in 2008–2009, there were >38,600 estimated cases of salmonellosis and 11,300 patients presenting to a primary care physician [2]. Hospital outbreaks of *Salmonella* may result from patient-to-patient spread and can be lethal in vulnerable patients [3–5]. An example is the hospital outbreak at Stanley Royd Hospital in the UK which led to the deaths of 19 patients and a public inquiry [2, 6]. We recently detected a cluster of more than 30 cases of *Salmonella enterica* serovar Enteritidis (*S.* Enteritidis) over a 3-week period at one of three hospital sites in our hospital organisation and from the community. This was against a typical background incidence of five to eight cases per month of all *S. enterica* isolates in the area served by our hospital. Initially a small number of seemingly unrelated, presumed community-acquired cases were detected on different wards but subsequently a larger number of long-term inpatients on two adjoining wards were affected suggesting the possibility of spread within the hospital. Simultaneously, an increase in community isolates was also detected. At first, it was unclear whether hospital cases were reflecting multiple imports from a community outbreak or spread within the hospital or both. Due to the explosive nature of the outbreak, coupled with uncertainty about the source, a rapid response was required to ensure that infection control measures were appropriately targeted. Outbreak investigations are aided by rapid availability of whole-genome sequencing (WGS) data, as this provides the greatest level of discrimination between isolates when compared to traditional typing methods such as phage typing, multilocus variable number tandem repeat analysis (MLVA) and pulsed-field gel electrophoresis (PFGE) [3–5, 7]. The Illumina MiSeq sequencing platform has emerged as the gold standard for WGS investigations of outbreaks, but results may not be available for as long as 3 working days, depending on the protocol used [8–10]. A number of studies have evaluated the utility of WGS for typing *S. enterica* isolates; however, to the authors’ knowledge, this is the first use of prospective typing of this organism during an outbreak. Rapid availability of accurate typing results is critical to effective outbreak control. We therefore devised a novel rapid draft sequencing protocol on the MiSeq generating results in under 6 h following library preparation. At the time of the outbreak we were testing a portable, handheld, ‘USB stick’, whole-genome sequencer, the MinION (Oxford Nanopore Technologies, UK), as part of their early access programme. We wished to see what role this technology might play in the management of future outbreaks.

Our initial goals when performing sequencing prospectively were: (1) to determine if cases in the hospital were from the same strain as those circulating in the community, and to discriminate outbreak cases from normal background *S. enterica* strains; (2) to determine whether there was evidence of a super-shedder patient or specific breakdown in infection control practices; (3) to help link cases to a primary source (for example, person or food) and to compare to previous outbreak strains; and (4) to integrate these results with national surveillance data.

## Results and discussion

### Epidemiological investigation

**Fig. 1:**
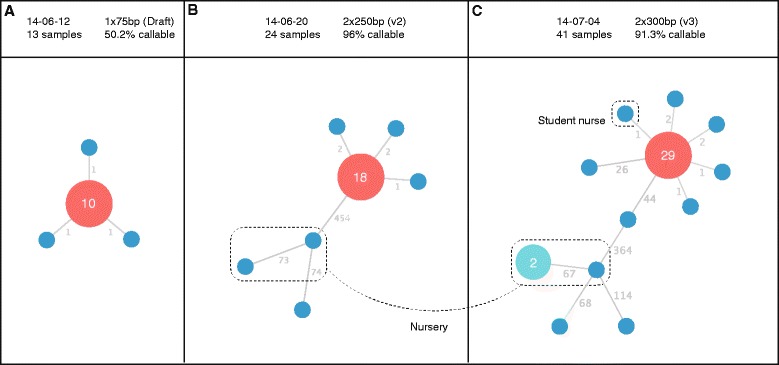
Phylogenetic reconstruction of the outbreak, as sequencing data were generated at three time points during the outbreak (corresponding to Panels **a, b** and **c**). The red node demonstrates the most frequently occurring SNP type. Node labels show the number of isolates of this type. Blue nodes are singletons. Edge labels show the number of SNPs between isolates. The turquoise node shows a cluster of two identical isolates from cases associated with a nursery. The header bar shows the date of the sequencing, the Illumina sequencing protocol used, the number of samples in the dataset and the total percentage of the *S.* Enteritidis reference genome which could be used for SNP calling (core genome). Subtle differences in the number of SNPs between nodes on the tree may be seen, for example in the unrelated nursery isolates (6 SNPs different between Panel **a** and Panel **b**). These differences are attributed to differences in core genome size, that is, the number of positions in the reference genome used to generate the results. The precise core genome size figures are shown as a percentage in the panel headings

In total, 43 isolates of *S.* Enteritidis were identified in the study period (1 to 24 June) from inpatients, community samples from general practitioners and from environmental isolates. Hospitalised cases were only identified at one hospital site in the group of three hospitals. The same hospital food is distributed to all three hospital sites from a single, central kitchen processing unit where hot food is twice-cooked to standards that would kill salmonellae. All microbiological testing of hospital food was negative for *Salmonella*. The environmental swabs from affected wards were all negative apart from one isolate of *S.* Enteritidis recovered from the outside door seal of a food regeneration trolley. This proved to be of the outbreak type. Four separate colony picks were sequenced from this culture. Isolates from staff were sent to the reference laboratory by another laboratory in a different city 14 miles away. These were detected in faecal samples submitted by general practitioners and were found to belong to staff at our hospital working on the affected wards. The first 16 samples, of which six cases had onset dates compatible with community acquisition, were available for sequencing on 10 June and 13 samples sequenced successfully. These were subsequently shown to be distinct from other isolates recently sequenced by national surveillance and were identical to each other, apart from three cases that each had one SNP difference (Fig. 1, Panel a).

Sequencing of isolates from two early patient cases on 3 and 4 June showed them to be identical. As both patients had been hospitalised for longer than the *Salmonella* incubation period this was strongly suggestive of hospital acquisition. There were nine other cases on or prior to 4 June, which together with the typing data helped to inform further infection control actions. All symptomatic patients were isolated and the two wards were closed. Deep cleaning was undertaken with vaporised hydrogen peroxide sterilisation. Four isolates from the later part of the outbreak were identified by SNP typing to be unrelated to the outbreak type. Two of the four isolates were from young children who had recently returned from separate holidays in Egypt. These isolates were different to each other but one was identical to another isolate from a child of similar age who had not travelled abroad, which prompted further epidemiological investigation. It emerged that the two children attended the same nursery in a town just outside the city in which the hospital outbreak had been detected, strongly suggesting transmission had occurred within the nursery (Fig. 1).

The earliest date of onset was 25 May and the last 8 July. A total of 37 cases with the outbreak strain were identified of which six had no connection with the hospital, eight were staff members and three were asymptomatic carriers identified by screening patients on outbreak wards. Comparison of the outbreak genome sequence with the Public Health England database of strains from the whole of England and Wales suggested a very close relationship to six isolates from London, Bedford and Northampton. Further epidemiological investigation of these cases found no link to Birmingham or a foodstuff. The outbreak strain was PT 14b and multi-locus variable number tandem repeat (MLVA) type 2-11-9-7-4-3-2-8-9, an uncommon type for PT 14b strains.

### Rapid draft sequencing on the Illumina MiSeq

**Fig. 2:**
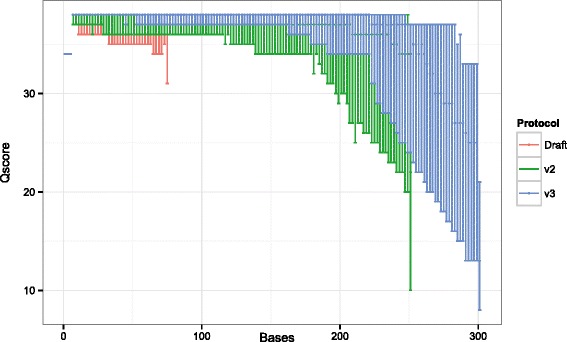
Phred-scaled quality scores (−10 log_10_ *P*) for Illumina sequencing demonstrating the impact of read length on read quality scores with the three Illumina MiSeq sequencing modalities used in this study. Red scores indicate results from the draft 1 × 75 base sequencing protocol, which shows minimally worse quality drop-off than running V2 (green, 2 × 250 base) or V3 chemistry (blue, 2 × 300) under standard conditions

As an initial response to the outbreak, isolates from 16 patients were sequenced overnight on the Illumina MiSeq on 12 June 2014 in order to generate results for an infection control meeting the following day (results shown in Fig. 1 Panel a). To enable this, we devised a new draft sequencing protocol that reduced the run time of the MiSeq instrument to 6 h (contrasted with standard protocols which can take up to 55 h to complete). This was achieved by reducing the read length, cycle time and number of tiles imaged. Of the 16 isolates, 13 had a mean coverage depth of greater than 4× (mean 8×) and could be used for further analysis. Due to the lower coverage of strains, 50.2 % of the core genome was used to generate these results. Despite this, the results generated within 6 h were sufficient to conclude that the initial set of isolates were all part of the same outbreak (10/13 isolates were identical when analysing the core genome of *S.* Enteritidis, three other isolates each differed by 1 SNP). Later on, when standard protocol MiSeq (paired 250 or 300 bp) data were available as well as HiSeq data from PHE surveillance, we were able to compare these results to that of draft sequencing. We could then conclude that although genome coverage was lower, the rapid draft sequencing method was concordant with both slower methods (Fig. 1). The sequencing quality using the draft protocol was lower (median Q score 36 compared with 38 using the V2 and V3 protocols at cycle 75) (Fig. 2).

### Retrospective evaluation of real-time nanopore sequencing

Two samples, one belonging to the outbreak and one unrelated were sequenced on the newly-available MinION from Oxford Nanopore Technologies. During the outbreak, we used an early version of the chemistry termed R6. However, results from this sequencing did not produce sufficient numbers of high-quality two-direction (2D) reads to be of use. In July 2014 R6 chemistry was replaced by R7, which we were able to evaluate retrospectively. The MinION is characterised by very long reads, which have a high error rate compared to the Illumina platform.

### Nanopore sequencing results

In order to evaluate the potential benefits of real-time sequencing to enhance infection control procedures we analysed read sets as they would have become available in real time, that is, at 10 min intervals after the run had been initiated. The two samples were run on separate flow cells. The number of reads generated in the first 170 min were 2,865 (first flowcell) and 3,447 (second flowcell) with mean read lengths of 6,340 and 4,664 bp, respectively for each sequence library. The mean read accuracy, determined by counting all differences from the reference genome, was 72 % (first flowcell) and 73 % (second flowcell).

### Real-time strain identification from nanopore reads

**Fig. 3:**
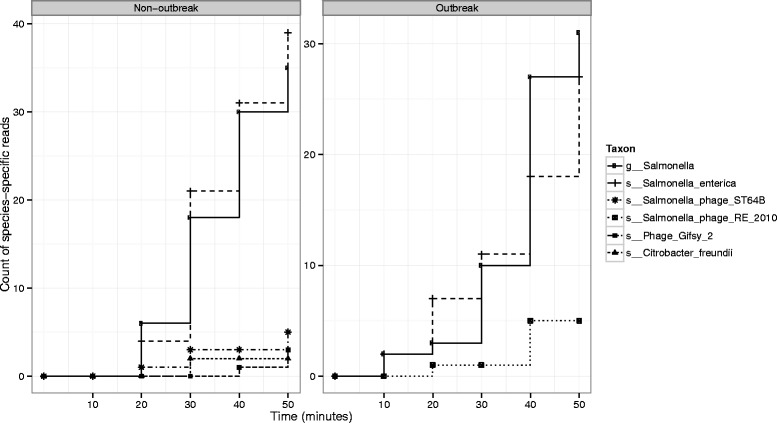
Streaming taxonomic assignments from the Oxford Nanopore MinION are shown for an isolate belonging to the outbreak and an isolate not belonging to the outbreak. Assignments to *Salmonella* and *Salmonella enterica* are found within 10 min of starting sequencing for the outbreak strain and within 20 min for the non-outbreak strain. Phage-specific sequences are detected and are distinct between the non-outbreak and outbreak strain. The non-outbreak strain harbours *Salmonella* phage ST64B and Gifsy-2, whereas the outbreak strain harbours *Salmonella* phage RE2010

We found that in the two samples tested we could unambiguously identify the bacterial species *S. enterica* in less than 30 min (Fig. 3). Additionally, chromosomally encoded phage sequences were detectable and distinguishable between outbreak and non-outbreak strains within 50 min.

### Genotyping from low coverage, error-prone data using phylogenetic placement

**Fig. 4:**
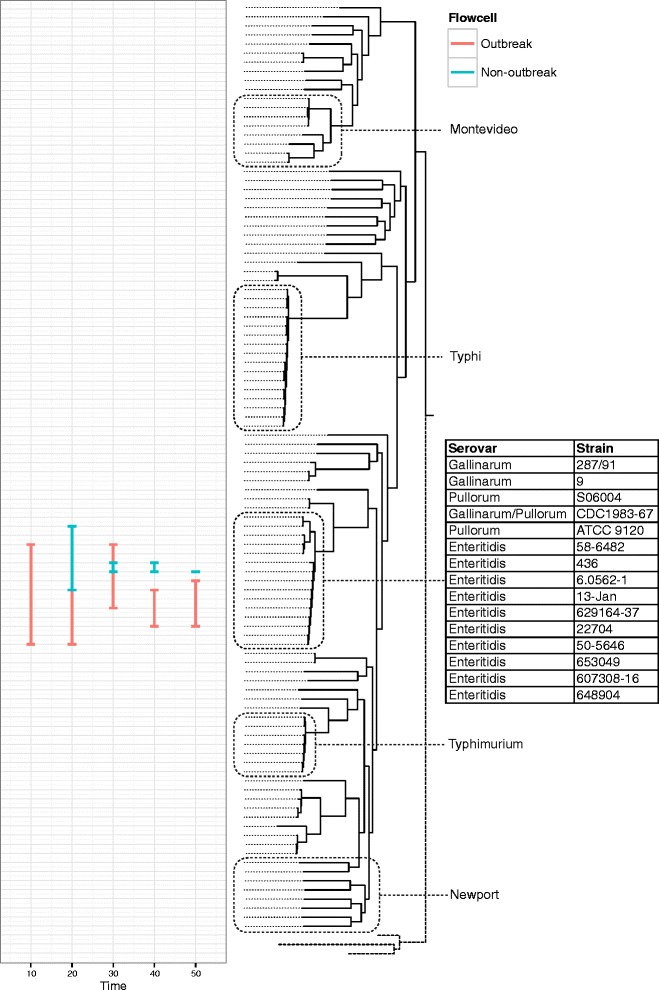
Results of streaming phylogenetic placement from the Oxford Nanopore MinION on a reference tree of representative published *Salmonella enterica* sequences. Common serovars of Salmonella are highlighted. Both outbreak and non-outbreak strains are unambiguously identified as *Salmonella enterica* serovar Enteritidis by their position on the phylogenetic tree within 50 min. The line demonstrates the potential range of placements reported by *pplacer*. The red placements indicate the positions of the outbreak isolate and the blue placements indicate the positions of the non-outbreak isolate

**Fig. 5:**
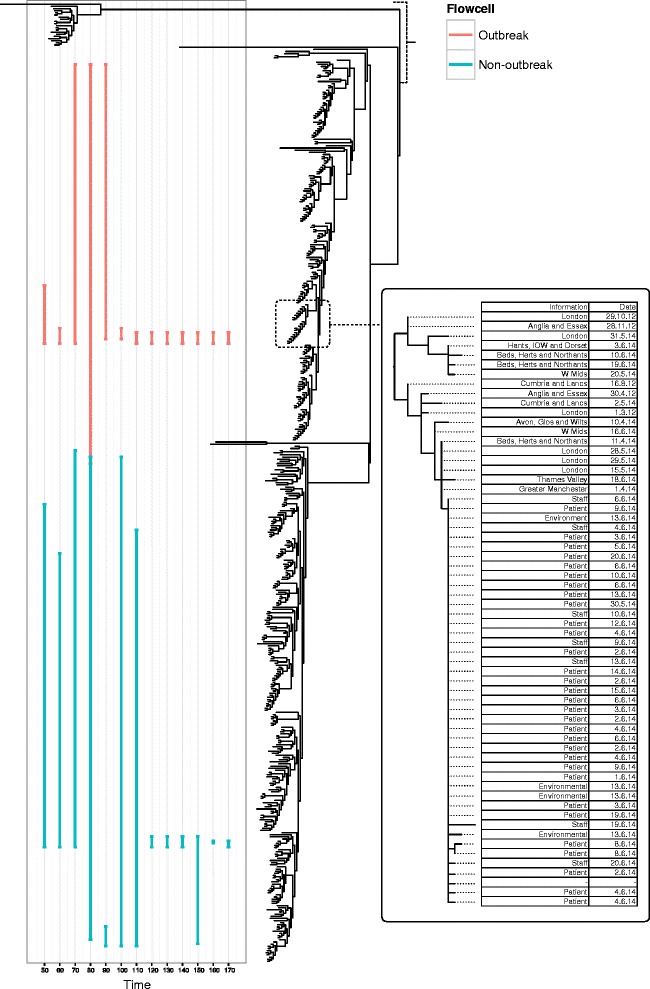
Results of streaming phylogenetic placement from the Oxford Nanopore MinION on a reference tree of *Salmonella enterica* serovar Enteritidis isolates collected by Public Health England during routine surveillance. The left-most panel demonstrates the confident placing of the outbreak isolate in the outbreak clade within 100 min, and the confident placing of the non-outbreak isolate into a clade containing multiple serotypes of *Salmonella* within 120 min. The red placements indicate the positions of the outbreak isolate and the blue placements indicate the positions of the non-outbreak isolate. The right-most panel shows a phylogenetic reconstruction of isolates from the outbreak and their source, set in context of a national outbreak of phage type 14b. Uncertainty in the phylogenetic placement technique is demonstrated early on in sequence data collection due to the low accuracy of the variant calls collected. As more data are collected, the number of possible phylogenetic placements reduces and the confidence values increase (not shown)

Genotyping accuracy improves as more sequencing data are available and a consensus sequence is formed (Table 1). Our genotyping protocol gets increasingly more precise as more reads are added, however recall stays relatively constant. Despite this, a phylogenetic placement method confidently assigned both the outbreak and non-outbreak strains to a clade of *S. enterica* containing the Gallinarum, Pullorum and Enteritidiis serovars very early on in the sequencing process. By 40 min it was possible to determine that the likely serovar was Enteritidis (Fig. 4). Once assigned to a serovar, further analysis could be restricted to a reference tree of *S.* Enteritidis strains. It was possible then to show that the outbreak strain unambiguously belonged to the main hospital outbreak cluster within 100 min of starting sequencing (Fig. 5). The non-outbreak strain was assignable to a clade containing several closely related strains (with a mixture of phage types, none of them PT 14b) within 120 min.

**Table 1:** Streaming alignment statistics from nanopore data

Flowcell	Time (m)	Reads	Bases	Positions	Missing bases	Covered (%)	True positive	True negative	False positive	False negative	Recall	Precision	Accuracy
Outbreak	60	920	5635627	7091	6463	8.86	10	617	0	2	0.83	1.00	0.09
Outbreak	120	2037	12853716	7091	4815	32.10	26	2237	7	7	0.79	0.79	0.32
Outbreak	180	3040	19297035	7091	3580	49.51	48	3436	13	15	0.76	0.79	0.49
Outbreak	240	3933	24900526	7091	2703	61.88	62	4291	17	19	0.77	0.78	0.61
Outbreak	300	4525	28614437	7091	2236	68.47	70	4736	25	25	0.74	0.74	0.68
Outbreak	360	5654	35848389	7091	1499	78.86	82	5454	26	31	0.73	0.76	0.78
Outbreak	420	6680	42498530	7091	1029	85.49	87	5914	25	37	0.70	0.78	0.85
Outbreak	480	7516	47950926	7091	749	89.44	94	6185	30	34	0.73	0.76	0.89
Outbreak	540	7913	50372188	7091	630	91.12	96	6300	29	37	0.72	0.77	0.90
Outbreak	600	8807	56254898	7091	463	93.47	103	6470	20	36	0.74	0.84	0.93
Outbreak	660	9666	61989423	7091	337	95.25	107	6588	22	38	0.74	0.83	0.94
Outbreak	720	10472	67171497	7091	267	96.23	111	6659	16	39	0.74	0.87	0.95
Outbreak	780	10833	69363106	7091	243	96.57	112	6686	16	35	0.76	0.88	0.96
Outbreak	840	11708	74625788	7091	191	97.31	117	6737	13	34	0.77	0.90	0.97
Outbreak	900	12479	79551399	7091	141	98.01	121	6780	16	34	0.78	0.88	0.97
Outbreak	960	13198	84228957	7091	120	98.31	124	6797	16	35	0.78	0.89	0.98
Outbreak	1020	13579	86600020	7091	107	98.49	125	6808	16	36	0.78	0.89	0.98
Outbreak	1080	14359	91437571	7091	90	98.73	126	6823	17	36	0.78	0.88	0.98
Outbreak	1140	15168	96646434	7091	74	98.96	124	6842	15	37	0.77	0.89	0.98
Outbreak	1200	15835	100970757	7091	70	99.01	123	6851	12	36	0.77	0.91	0.98
Outbreak	1260	16205	103367082	7091	63	99.11	124	6857	11	37	0.77	0.92	0.98
Outbreak	1320	16632	106040214	7091	60	99.15	125	6859	12	36	0.78	0.91	0.98
Outbreak	1380	17184	109618605	7091	56	99.21	125	6863	11	37	0.77	0.92	0.99
Outbreak	1440	17332	110500445	7091	55	99.22	124	6865	11	37	0.77	0.92	0.99
Non-outbreak	60	1268	5382184	7091	6372	10.14	1	717	2	0	1.00	0.33	0.10
Non-outbreak	120	2554	11567191	7091	4791	32.44	2	2284	15	0	1.00	0.12	0.32
Non-outbreak	180	3626	17058822	7091	3451	51.33	4	3607	29	1	0.80	0.12	0.51
Non-outbreak	240	4612	22004574	7091	2500	64.74	11	4545	32	4	0.73	0.26	0.64
Non-outbreak	300	5483	26582592	7091	1760	75.18	13	5281	35	3	0.81	0.27	0.75
Non-outbreak	360	6198	30340527	7091	1330	81.24	15	5705	40	2	0.88	0.27	0.81
Non-outbreak	420	6877	34040490	7091	985	86.11	16	6054	35	2	0.89	0.31	0.86
Non-outbreak	480	7522	37471113	7091	727	89.75	18	6306	37	4	0.82	0.33	0.89
Non-outbreak	540	8306	41387560	7091	552	92.22	18	6483	34	5	0.78	0.35	0.92
Non-outbreak	600	9032	45052523	7091	395	94.43	20	6643	28	6	0.77	0.42	0.94
Non-outbreak	660	9682	48325820	7091	304	95.71	20	6735	27	6	0.77	0.43	0.95
Non-outbreak	720	10262	51312827	7091	262	96.31	20	6783	21	6	0.77	0.49	0.96
Non-outbreak	780	10845	54417219	7091	202	97.15	21	6845	18	6	0.78	0.54	0.97
Non-outbreak	840	11346	57135819	7091	178	97.49	22	6870	16	6	0.79	0.58	0.97
Non-outbreak	900	11793	59514439	7091	145	97.96	23	6898	18	8	0.74	0.56	0.98
Non-outbreak	960	12192	61590631	7091	111	98.43	22	6932	19	8	0.73	0.54	0.98
Non-outbreak	1020	12571	63597395	7091	99	98.60	22	6944	19	8	0.73	0.54	0.98
Non-outbreak	1080	12926	65415215	7091	87	98.77	21	6959	18	7	0.75	0.54	0.98
Non-outbreak	1140	13263	67138579	7091	71	99.00	22	6976	15	8	0.73	0.59	0.99
Non-outbreak	1200	13594	68911549	7091	62	99.13	22	6985	15	8	0.73	0.59	0.99
Non-outbreak	1260	13881	70408443	7091	59	99.17	22	6992	11	8	0.73	0.67	0.99
Non-outbreak	1320	14186	72080944	7091	53	99.25	23	7001	8	7	0.77	0.74	0.99
Non-outbreak	1380	14471	73573256	7091	44	99.38	23	7008	10	7	0.77	0.70	0.99
Non-outbreak	1440	14683	74801565	7091	40	99.44	23	7012	10	7	0.77	0.70	0.99

The columns show (from left to right): (1) the sample analysed; (2) the cumulative results at this time period (min); (3) the total number of two-direction reads; (4) the total number of nucleotide bases; (5) the total size of the alignment; (6) the number of bases in the alignment missing from the dataset; (7) the percentage of bases in the alignment that can be called; (8) the count of true positives; (9) the count of true negatives; (10) the count of false positives; (11) the count of false negatives; (12) the recall, that is, sensitivity, calculated as TP/(TP + FN); (13) the precision, calculated as TP/(TP + FP); (14) the accuracy, calculated as (TP + TN)/(P + N)

The availability of definitive typing data so early on in this outbreak enabled us to identify transmission between hospital wards and take rapid action to control spread. The appearance of cases in unrelated wards was puzzling initially, but WGS confirmed that the hospital SNP type was the same as that circulating in the community. This reassured the infection control team that there was not hospital-wide spread via some unknown vector. Preliminary food sample testing results were only available one day later. The finding of the outbreak strain on the door seal of the food trolley with the subsequent confirmation of cases in staff members supported the hypothesis that some local spread had occurred via the environment. Person-to-person spread may also have occurred. Towards the end of the outbreak the ability to rapidly identify cases not involved prevented much wastage of effort and resources. Remarkably we identified transmission of another strain of *S.* Enteriditis probably acquired in Egypt in a childcare group at a distant site because of the resolution of the typing information directing epidemiological investigations. Recent outbreaks of PT 14b strains in the UK have previously been associated with Spanish eggs, although the antibiotic resistance profile of the outbreak described here is different [11, 12]. Contemporaneously, outbreaks of *S.* Enteritidis PT 14b associated with consumption of eggs were reported in France, Austria and Germany, triggering an urgent outbreak investigation by the ECDC and EFSA [12]. Strains associated with this outbreak were of MLVA type 2-12-7-3-2 (using the 5-locus scheme), varying by a single locus from the isolates identified in this study. In these cases *S.* Enteritidis was isolated from eggs originating from a producer in Germany [12]. There is no definitive link between the outbreak reported in this study and the consumption of German eggs. However, the MLVA type in the European outbreak was also detected in the UK and eggs from the German producer are distributed for sale in the UK. Further whole-genome sequencing of European isolates is now being undertaken and may help determine whether the two outbreaks are linked to a common source.

This study illustrates a substantial future benefit from extremely rapid definitive WGS typing. The epidemiology of non-typhoidal *Salmonella* has changed significantly in the UK over the last decade and to a lesser extent in the rest of Europe [2, 13]. While non-typhoidal *Salmonella* rates have fallen overall, particularly in the UK following chicken flock vaccination, the proportion of disease caused by *S.* Enteritidis associated with travel has risen greatly. The ability to both identify serovars via deduced multi-locus sequence typing (MLST) and specific strains within a day of bacterial colonies being available will enable outbreaks to be investigated at a stage where accurate travel/food histories and possible person-to-person transmission can be elucidated and control measures introduced. We show that our method of rapid draft sequencing on the MiSeq is able to generate reliable results, despite generating reduced genome coverage. We anticipate this method will be of value to research groups needing to generate results in the timescale of a single working day, a considerable reduction compared to the standard protocols on this instrument.

The availability of national and international databases of sequencing data of food-borne pathogens marks an exciting step forward for epidemiological investigations. Surveillance by WGS has been pioneered by the US Food and Drug Administration, with results published online on the National Center for Biotechnology Information’s GenomeTrakr service, an advantage of the portable, digital nature of genome data [8, 14]. In the UK, since 1 April 2014, Public Health England has been routinely sequencing all *Salmonella enterica* strains reported by hospitals and general practitioners to the *Salmonella* Reference Service, Colindale. Through integration with this dataset, we determined that the outbreak strains formed a distinct cluster, although this cluster varied by only a single core SNP from cases observed elsewhere in the UK.

We evaluated two sequencing methodologies in this study, both capable of providing rapid whole-genome sequencing information. The MinION senses individual DNA strands as they move through a protein nanopore. A unique property of this technology is that sequence data are available in real time, and analysis can be performed on a continuous stream of long reads. We wished to evaluate the potential impact of a real-time approach for analysis of clinical bacterial isolates. We exploited this feature to perform rapid identification and typing of genomic DNA prepared from a pure colony isolate. Given the high error rate reads generated in this study we employed a database of taxon-defining genes from microbial species to make bacterial and bacteriophage identifications [15]. This approach is tolerant of low-coverage, high-error reads making it useful for real-time analysis of nanopore sequences. However, due to the higher error rate of this platform, a *de novo* SNP calling approach as utilised with MiSeq data would not produce informative results within the short time scales of interest here. Other studies have investigated the error rate and mode of this instrument in greater detail [15, 16, 17]. We show that despite the high error rate, effective genotyping is possible using phylogenetic placement techniques. Phylogenetic placement has been used to good effect in metagenomics studies where only low-coverage data are available, for example in the diagnosis of infectious diseases from ancient DNA samples, directly from sputum and from the hospital environment [18–20]. Using this approach, and a simple heuristic algorithm to call the most likely genotype it was possible to reliably place streaming nanopore data onto a reference phylogeny despite the high read error rate. Other studies have shown that genotyping accuracy can reach 99 % when very high coverage (>120×) is available. This would permit a *de novo* genotyping approach which did not rely on phylogenetic placement, as is more typical in studies employing traditional high-throughput sequencing [14].

**Fig. 6:**
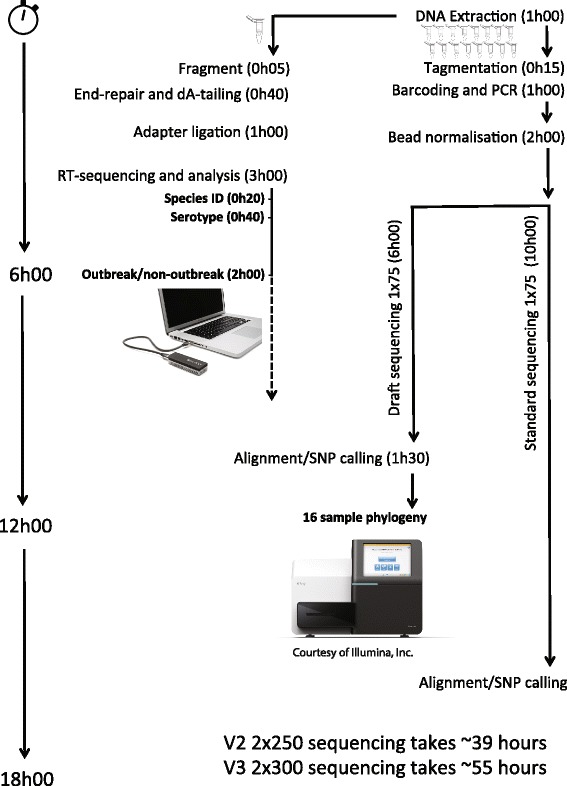
A schematic showing time-to-result for the sequencing analysis strategies used in this study, starting from DNA input

Both the draft sequencing protocol presented for the MiSeq and the real-time evaluation of nanopore sequencing demonstrate that these approaches have utility for generating data of use in outbreak investigations in less than one day (Fig. 6). It is not our intention here to perform direct comparisons between the instruments in this study, particularly as they are quite different in their mode of operation.

The MiSeq is typically run in a factory-style ‘batch’ mode, where many bacterial samples (up to 100 on a MiSeq, or potentially many hundreds on the larger HiSeq instrument) are run simultaneously, and processed in serial at the end of the instrument operation. This approach reduces the cost of sequencing by taking advantage of the very high output offered from these instruments (>1 terabase for the HiSeq in High Output Mode). The precipitous drop in the cost of sequencing bases has meant that for bacterial applications the cost of library preparation is rapidly becoming the most expensive component. However, batch methods, particularly with the very highest output modes result in a flexibility trade-off; such an approach means that data cannot be analysed until at least the barcode identifiers have been read (usually not until after halfway through the run).

This is in contrast to the real-time sequencing approach of the MinION whereby individual samples are loaded, and results are generated and analysed in real time until the results are sufficient to address the clinical question. Such an approach has appealing properties for applications such as infectious disease diagnostics. A second attribute of the MinION that is notable is its extreme portability, comparable in size to a USB flash drive and requiring only a basic laptop to draw power from and connect to. This suggests that it may, in principle, be possible in the future to move sequencing closer to the sample, and particularly when coupled with a culture-independent approach.

However, at present the instrument depends on access to a basic molecular biology laboratory infrastructure, including access to freezer, and basic laboratory equipment such as heater blocks and pipettes. The existing library preparation method, although relatively quick, is quite labour-intensive for each sample. Presently there is no method for multiplexing large numbers of bacterial genomes (as with the MiSeq instrument), nor would the throughput be amenable to this. Therefore, it seems likely for large-scale surveillance efforts this platform is not the obvious choice, for reasons of labour and cost. Instead, we envisage that development of rapid library preparation assays will be necessary in order to see this platform become usable in a clinical microbiology laboratory or patient setting in the manner described here.

Furthermore, the need for culture enrichment remains a significant bottleneck for rapid identification of bacteria and this also applies to other studies employing whole-genome sequencing. Culturing of *Salmonella* takes between 24 h (presumptive diagnosis) and 48 h (pure culture for sequencing). Our approach, which relies on sequencing single colonies from each sample, is a limitation of this and similar studies. However, sequencing of four individual colonies from the food trolley demonstrated very limited heterogeneity with three isolates being identical to the majority of other cases in the outbreak, and one showing two SNP differences. A culture-free approach for bacterial diagnostics has been recently proposed and this would permit detection of mixed infections as well as cutting down the time to result significantly, for example in the case of direct sequencing of Shiga-toxin producing *E. coli* from stool samples and *M. tuberculosis* directly from sputum [21, 22]. However, sequencing mixed communities reduces the genomic coverage of the pathogenic target of interest, and so for such an approach to be successful it is likely to rely on generating greater throughput than currently achievable on the MinION. Enrichment for the target organism, most easily attained through traditional microbiology culture, is therefore still a required stage.

## Conclusion

The combination of rapid prospective sequencing during an outbreak and detailed characterisation of cases occurring on a national scale has potential implications for the future of outbreak investigation [23]. We describe a novel protocol for draft sequencing on the MiSeq that is sufficiently quick to determine whether an outbreak is occurring. For this vision to become a reality, further work is needed to enable sharing of data between hospitals and community practitioners with public health laboratories. Larger scale integration with national genome databases represents the first implementation of a new paradigm for the investigation of outbreaks. The use of rapid, draft sequencing can delineate the context of an outbreak very quickly even at lower than usual genome coverage.

## Materials and methods

### Sample and bacterial culture collection

Faeces samples from patients with diarrhoea were submitted for culture and plated on XLD medium. Presumptive *S. enterica* isolates were confirmed using biochemical tests and O- and H-antigen agglutination sera and all those identified as *S.* Enteritidis were retained for molecular typing. Environmental swabs were taken from the affected wards within 24 h of the ward clusters being identified and were processed as above.

### Genomic DNA extraction

Genomic DNA was prepared from nutrient agar slopes incubated for 4–18 h at 37 °C. Cells were harvested using 100 μL of sterile PBS added to the surface and a sterile loop used to emulsify bacteria into a suspension that was then pipetted into a sterile Eppendorf tube. This suspension was used to harvest DNA with the ‘Invisorb spin cell mini kit’ (Invitek, Germany) according to the manufacturer’s instructions. The quantity of DNA in each sample was determined using a Qubit 2.0 fluorometer and dsDNA HS assay (Life Technologies, Paisley, UK).

### Library preparation for Illumina MiSeq sequencing

Sequence-ready libraries were generated from 1 ng DNA per sample using the Nextera XT library preparation kit (Illumina, Great Chesterford, UK) according to the manufacturer’s instructions.

### Rapid draft sequencing on the Illumina MiSeq

In order to provide results for an emergency infection control meeting the next morning, we adapted the standard sequencing protocol on the Illumina MiSeq to rapidly generate sufficient data to analyse 16 strains. We utilised a standard *V3* 600-cycle reagent kit. By modifying the recipe files on the instrument we reduced the chemistry time by 40 s per cycle and the number of tiles imaged by 50 %. This resulted in a cycle time of approximately 3 min per cycle and allowed 75 base single-read sequencing with dual barcoding to complete within 6 h. We chose 75 base reads as a trade-off between expected genome coverage and available time in order to have results available sufficiently quickly for analysis. The sequencing protocol can be downloaded from [24].

### Standard sequencing on Illumina MiSeq and HiSeq

Later in the outbreak isolates were sequenced using the Illumina MiSeq with standard V3 protocol at the University of Birmingham, UK, also prepared with Nextera XT reagents. In addition, some outbreak isolates were sequenced on the Illumina HiSeq 2500 with TruSeq V3 reagents as part of the Public Health England (PHE) WGS sequencing pipeline at Colindale, UK (Additional file 1: Table S1).

### Phylogenetic reconstruction from draft sequencing

Before being mapped against the reference genome *S.* Enteritidis P125109 (PRJNA59247) with BWA-MEM (version 0.7.5), 75 base single-read data generated by draft sequencing on the Illumina MiSeq was adapter and quality trimmed with Trimmomatic [25, 26]. Single nucleotide polymorphisms (SNPs) were called using samtools mpileup (version 0.1.18) and VarScan (version 2.3.6), specifying a minimum read depth of 2 [27, 28]. Filtered SNPs (those positions with an allele frequency of >80 % to call a variant or <20 % to call the reference base in all samples) were extracted to make a concatenated FASTA alignment. FastTree (version 2.1.7) was used to generate an approximate maximum likelihood phylogenetic tree [29]. Phyloviz was used to produce minimum spanning tree reconstructions [30]. Functional annotation of these variants was performed using snpEff (version 3.1) [31].

### Phylogenetic reconstruction from PHE surveillance sequencing

Before being mapped against the reference genome *S.* Enteritidis AM933172 (PRJEA30687) with BWA-MEM, 100 base pair paired-end data generated on the Illumina HiSeq 2500 was adapter and quality trimmed [26]. SNPs were called using GATK [32]. High quality SNPs (>10-fold coverage, >30 mapping quality, 90 % consensus) were selected and uploaded into SNPdatabase (SNPdb). This is an in-house PostgreSQL database containing genome position and variant base for each SNP and low quality/missing positions for all *S.* Enteritidis eBURST group 4 (EBG 4) isolates sequenced by PHE. SNPs in the core genome of the strain set being analysed were extracted from an in-house SNPdb and FastTree was used to derive approximate maximum likelihood phylogenetic trees. Annotation data came from the in-house PHE GastroDataWarehouse (GDW).

Due to the clinical interest in these cases, strains with below standard sequencing depth (30×) were analysed and this had no impact on the analysis outcome, with identical tree topologies detected in all cases.

### Real-time sequencing on the MinION

An outbreak and a non-outbreak isolate, as determined by earlier MiSeq sequencing, were chosen for sequencing on the MinION (Oxford Nanopore Technologies, Oxford, UK) to assess its suitability for future outbreak investigations. High-molecular weight input DNA (1 μg) was fragmented using a Covaris G-Tube (Covaris, Woburn, USA) at 5,000 rpm in a centrifuge. Fragmented DNA was end-repaired using the NEB repair module (New England Biolabs, Ipswich, USA), then cleaned-up using SPRI beads with a ratio of 1:1 beads to reaction mixture. End-repaired DNA was then A-tailed using the NEB A-tailing module. Following this a sequence-ready library was generated using the gDNA sequencing kit and protocol provided as part of the MinION access program (MAP). The diluted library (150 μL) was loaded into the MinION flowcell via the sample loading port. A 72-h sequencing protocol was initiated using the MinION control software, MinKNOW (version 0.45.2.6). Read event data were base-called by the software Metrichor (version 0.16.37960) using workflow 1.0.2_R7. The FASTA sequences and strand translocation times were extracted for further analysis using the poretools FASTA extraction function [33]. All sequence data are deposited into the Short Read Archive (SRA) with study reference ERP006904 (MinION data) and ERP007194 (Illumina data).

### Species identification from nanopore reads

Identification of bacterial and viral species present in each sample was carried out using an alignment method to the MetaPhlAn 2 database of taxon-defining marker genes [33]. First, the database was extracted into FASTA format using the fastacmd utility supplied with NCBI BLAST. Alignment of nanopore reads was performed using the LAST package (version 475), invoking lastal with custom settings as per Quick *et al.* [15], using a gap creation penalty and extension of 1 and a mismatch penalty of 2 (match score 1), corresponding to command line arguments -a1 -b1 -q2.

### Subspecies level classification from nanopore reads

Serovars of *S. enterica* can often be assigned by phylogenetic methods. A phylogenetic reference tree was created from the available draft or complete *Salmonella enterica* genomes in RefSeq. From each of the sequences 600,000 simulated paired-end reads were generated using wgsim (version 0.3.1) [34]. These were mapped against the reference genome *S.* Typhimurium LT2 (PRJNA57799) with BWA-MEM (version 0.7.5) [26]. samtools mpileup (version 0.1.18) and VarScan (version 2.3.6) were used to call variants [27, 28]. Variant filtering was done using filter_non_discriminatory_variants.py [35] in order to remove non-discriminatory positions, as well as heterozygous positions and regions of putative recombination. Variant alleles for each sample were concatenated into a fasta file using vcf2phyloviz.py [36]. This file was de-duplicated using the mogrify command in seqmagick (version 0.6.0) to remove identical sequences which can affect placements. A phylogenetic reconstruction was created using FastTree (version 2.1.7) following a generalised time reversible model, after which taxtastic (version 0.5.1) was used to build the reference package [37].

To determine the subspecies level classification from the nanopore sequencing data, the reads were mapped against the reference genome with lastal with settings -a1 -b1 -q2. For each read, the highest scoring alignment was taken before being converted into BAM format using samtools. Using samtools mpileup and the script get_alleles_from_pileup.py the alignment was interrogated at all coordinates used for the reference tree. Aligned bases at these coordinates were counted and the dominant allele was used if at least two concordant bases were in the alignment. Alleles were concatenated into an alignment. Gap characters were used to represent uncertain positions not meeting the above criteria. The phylogenetic placement utility, pplacer, was used to place the sequence onto the reference tree producing a file containing the most likely position and logML probability for this placement. Placements with a likelihood value of greater than −500 were excluded [37]. This placement process was repeated for the read dataset available at each timepoint (10 min apart). New reads generated during each 10 min time interval were mapped to the reference, converted to a BAM file and merged with the BAM file generated at the previous time period.

### *S. enterica* outbreak reconstruction

As with the subspecies level classification, phylogenetic placement can be used as a method for classifying samples in or out of an ongoing outbreak and in a national and international context. In order to do this, we leveraged the routine surveillance sequencing of *S. enterica* by PHE using 575 *S.* Enteritidis genomes of phage type 14b. Using the method described above a phylogenetic reference tree was created for these genomes (448 remained after de-duplication) before the nanopore sequences were placed onto the tree to predict whether or not they belonged to the outbreak cluster.

## Additional file

Additional file 1: Table S1. Isolate identifiers sequenced in this study using rapid draft MiSeq sequencing, standard MiSeq sequencing and HiSeq sequencing during routine PHE surveillance.
