# Supplementary material for: Rapid draft sequencing and real-time nanopore sequencing in a hospital outbreak of Salmonella
Source: Genome Biol. 2015 May 30;16:114. doi: 10.1186/s13059-015-0677-2 (PMC4702336; doi:10.1186/s13059-015-0677-2)
Supplement: Additional file 1: Table S1. — Isolate identifiers sequenced in this study using rapid draft MiSeq sequencing, standard MiSeq sequencing and HiSeq sequencing during routine PHE surveillance. [file 13059_2015_677_MOESM1_ESM.pdf]

Date	12/06/2014	20/06/2014	04/07/2014	Surveillance
Plate	27	28	30	-
Format	1x75	250x250	300x300	100x100
Samples			907828	
	112797	112797		20909_H14242081901-1
		906014		
	907378	907378		18554_H14238030801-1
	907452	907452		21777_H14246029605-2/21781_H14246030005-2
		907462		18556_H14238031001-1/21785_H14246030505-2
	907474	907474		
	907479	907479		21778_H14246029705-2/21782_H14246030105-2
		907513		
	907525	907525		21786_H14246030605-2/21779_H14246029805-2
	907555	907555		21115_H14242077501-2
	907568	907568		21117_H14242078101-2
	907603	907603		21767_H14242077701-2
	907608	907608		21116_H14242077601-2
		907638		18555_H14238030901-1
	907648	907648		21114_H14242077401-2
		907659		21783_H14246030205-2
	907678	907678		21113_H14242077301-2
	907724	907724		21770_H14244069001-2
	907757	907757		20925_H14244069101-1
		907774		
	907789	907789		20926_H14244069201-1
	907812	907812		20927_H14244069301-1
		907826		
		907926	907926	21845_H14252045205-2
		907928		
		907946	907946	21844_H14252045105-2

907985	907985	21816_H14248028305-2
908004	908004	21843_H14252045005-2
908108	908108	
908127	908127	
	908151	
	908175	
	908346	
	908390	
	908397	
	908430	
	908445	
	908735	
	908736	
	908767	
	H142480277	
	H142580393	
	H142580394	
		21846_H14252045305-2
		21815_H14248028205-2
		21807_H14248027405-2
		21810_H14248027705-2
		21809_H14248027605-2
		21808_H14248027505-2

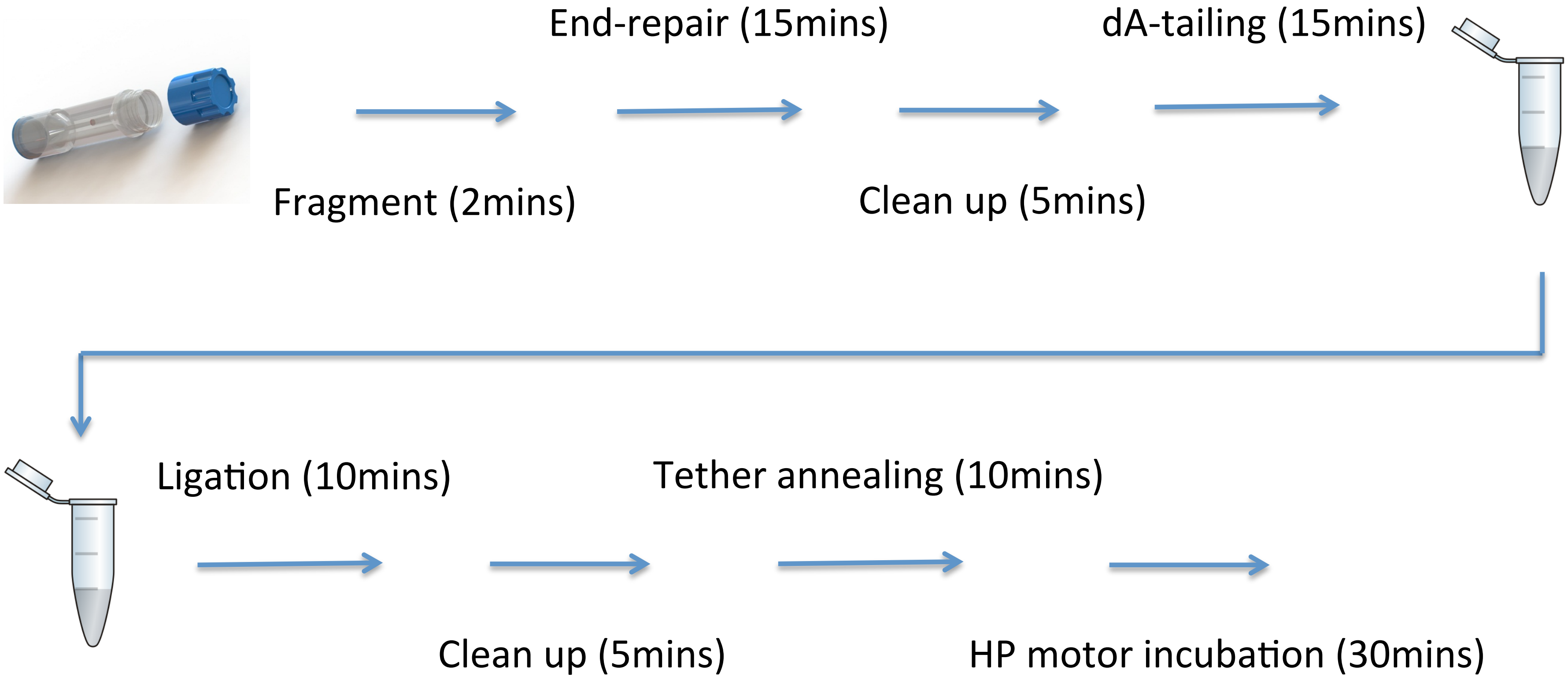

Total: 92 minutes
